# Supplementary material for: Recent Advances and Future Prospects in Immune Checkpoint (ICI)-Based Combination Therapy for Advanced HCC
Source: Cancers (Basel). 2021 Apr 18;13(8):1949. doi: 10.3390/cancers13081949 (PMC8072916; doi:10.3390/cancers13081949)
Supplement: Supplementary file 1 [file cancers-13-01949-s001.zip › cancers-1191817-supplementary.pdf]

Recent Advances and Future Prospects in Immune Checkpoint (ICI)-Based Combination Therapy for Advanced HCC

Yawen Dong, Jeffrey Sum Lung Wong, Ryohichi Sugimura, Ka-On Lam, Bryan Li, Gerry Gin Wai Kwok, Roland Leung, Joanne Wing Yan Chiu, Tan To Cheung and Thomas Yau

Table S1. Overview of exclusion criteria for each ICI-based combination study in advanced HCC.

Combination regimens	NCT number/ trial name	Exclusion criteria	
Durvalumab + tremelimumab	HIMALAYA (NCT03298451)	Hepatic encephalopathy within past 12 months or requirement for medication to prevent or control encephalopathy<br>Clinically meaningful ascites<br>Main portal vein tumor thrombosis<br>Active or prior documented GI bleeding (eg, esophageal varices or ulcer bleeding) within 12 months<br>HBV and HVC co-infection, or HBV and Hep D co-infection	[35]
Durvalumab + tremelimumab/ bevacizumab	Study 22 (NCT02519348)	Prior exposure to immune-mediated therapy<br>Hepatic encephalopathy within past 12 months or requirement for medications to prevent or control encephalopathy<br>GI Bleeding (eg, esophageal varices or ulcer bleeding) within 12 months<br>Ascites requiring non-pharmacologic intervention (eg, paracentesis) to maintain symptomatic control, within 6 months prior to the first scheduled dose.<br>Main portal vein thrombosis (Vp4) as documented on imaging<br>Any concurrent chemotherapy, immunotherapy, or biologic or hormonal therapy for cancer treatment<br>Active or prior documented autoimmune or inflammatory disease with some exceptions<br>Current or prior use of immunosuppressive medication within 14 days with some exceptions	[33]
Ipilimumab + nivolumab	CheckMate 040 (NCT01658878)	History of autoimmune disease<br>Any prior or current clinically significant ascites<br>Any history of hepatic encephalopathy	[30]
Ipilimumab + nivolumab	CheckMate 9DW (NCT04039607)	Known fibrolamellar HCC, sarcomatoid HCC, or mixed cholangiocarcinoma and HCC<br>Prior liver transplant<br>Episodes of hepatic encephalopathy (greater than or equal to [>=] Grade 2) within 12 months prior to randomization<br>Active brain metastases or leptomeningeal metastases	[34]
Atezolizumab + bevacizumab	IMbrave 150 (NCT03434379)	History of leptomeningeal disease<br>Active or history of autoimmune disease or immune deficiency	[46]

		History of idiopathic pulmonary fibrosis, organizing pneumonia, drug-induced pneumonitis, or idiopathic pneumonitis, or evidence of active pneumonitis on screening chest computed tomography scan<br>Known active tuberculosis<br>History of malignancy other than HCC within 5 years prior to screening, with the exception of malignancies with a negligible risk of metastasis or death<br>Pregnancy or breastfeeding, or intention of becoming pregnant during study treatment or within at least 5 months after the last dose of atezolizumab, 6 months after the last dose of bevacizumab, or 1 month after the last dose of sorafenib<br>Known fibrolamellar HCC, sarcomatoid HCC, or mixed cholangiocarcinoma and HCC<br>Untreated or incompletely treated esophageal and/or gastric varices with bleeding or high-risk for bleeding<br>A prior bleeding event due to esophageal and/or gastric varices within 6 months prior to initiation of study treatment.<br>Moderate or severe ascites<br>History of hepatic encephalopathy<br>Co-infection of HBV and HCV<br>Symptomatic, untreated, or actively progressing central nervous system (CNS) metastases<br>Uncontrolled tumor-related pain<br>Uncontrolled pleural effusion, pericardial effusion, or ascites requiring recurrent drainage procedures<br>Uncontrolled or symptomatic hypercalcemia<br>Treatment with systemic immunostimulatory agents<br>Inadequately controlled arterial hypertension<br>Prior history of hypertensive crisis or hypertensive encephalopathy<br>Evidence of bleeding diathesis or significant coagulopathy<br>History of intestinal obstruction and/or clinical signs or symptoms of GI obstruction including sub-occlusive disease related to the underlying disease or requirement for routine parenteral hydration<br>Serious, non-healing or dehiscing wound, active ulcer, or untreated bone fracture<br>Metastatic disease that involves major airways or blood vessels, or centrally located mediastinal tumor masses<br>Local therapy to liver within 28 days prior to initiation of study treatment or non-recovery from side effects of any such procedure<br>Chronic daily treatment with a non-steroidal anti-inflammatory drug (NSAID)	
Lenvatinib + pembrolizumab	KEYNOTE-524 (NCT03006926)	Prior treatment with lenvatinib or any anti-PD-1, anti-PD-L1, or anti-PD-L2 agent<br>Active malignancy (except for HCC or definitively treated melanoma in-situ, basal or squamous cell carcinoma of the skin, or carcinoma in-situ of the cervix) within the past 36 months	[48]

		Any medical or other condition which, in the opinion of the investigator, would preclude participation in a clinical trial<br>Active infection (any infection requiring systemic treatment). Hepatitis B or C [HBV/HCV] is allowed<br>Participants with CNS metastases are not eligible, unless they have completed local therapy (eg, whole brain radiation therapy [WBRT], surgery or radiosurgery) and have discontinued the use of corticosteroids for this indication for at least 4 weeks before starting treatment in this study. Any signs (eg, radiologic) or symptoms of brain metastases must be stable for at least 4 weeks before starting study treatment	
Lenvatinib + pembrolizumab	LEAP-002 (NCT03713593)	Has had esophageal or gastric variceal bleeding within the last 6 months<br>Has gastrointestinal malabsorption, gastrointestinal anastomosis, or any other condition that might affect the absorption of lenvatinib<br>Has a preexisting Grade ≥3 gastrointestinal or non-gastrointestinal fistula<br>Has clinically significant hemoptysis from any source or tumor bleeding within 2 weeks prior to the first dose of study intervention<br>Has significant cardiovascular impairment within 12 months of the first dose of study intervention such as history of congestive heart failure greater than New York Heart Association (NYHA) Class II, unstable angina, myocardial infarction or cerebrovascular accident stroke, or cardiac arrhythmia associated with hemodynamic instability<br>Has had major surgery to the liver within 4 weeks prior to the first dose of study intervention<br>Has had a minor surgery (ie, simple excision) within 7 days prior to the first dose of study intervention<br>Has serious non-healing wound, ulcer, or bone fracture<br>Has received any systemic chemotherapy for HCC or chemotherapy for any malignancy in the past 3 years<br>Has received prior therapy with an anti-programmed cell death 1 (ant-PD-1), anti-programmed cell death ligand 1 (anti-PD-L1), or anti- programmed cell death ligand 2 (anti-PD-L2) agent or with an agent directed to another stimulatory or co-inhibitory T-cell receptor (eg, cytotoxic T-lymphocyte-associated protein 4 [CTLA-4], OX-40, or CD137)<br>Has a diagnosis of immunodeficiency or is receiving chronic systemic steroid therapy or any other form of immunosuppressive therapy within 7 days prior the first dose of study intervention<br>Has a known additional malignancy that is progressing or has required active treatment within the past 3 years with the exceptions of basal cell carcinoma of the skin, squamous cell carcinoma of the skin, or carcinoma in situ (eg, breast carcinoma, cervical cancer in situ) that has undergone potentially curative therapy	[52]

		Has a known history of, or any evidence of, central nervous system (CNS) metastases and/or carcinomatous meningitis as assessed by local site investigator<br>Has severe hypersensitivity (≥Grade 3) to study intervention and/or any of their excipients<br>Has an active autoimmune disease that has required systemic treatment in past 2 years<br>Has a history of (non-infectious) pneumonitis that required steroids or has current pneumonitis<br>Has urine protein ≥1 grams/24 hours<br>Prolongation of corrected QT (QTc) interval to >480 milliseconds (corrected by Fridericia Formula)<br>Has left ventricular ejection fraction (LVEF) below the institutional normal range as determined by multigated acquisition scan (MUGA) or echocardiogram (ECHO)<br>Has an active infection requiring systemic therapy with the exceptions of hepatitis B virus (HBV) or hepatitis C virus (HCV)<br>Has a known history of human immunodeficiency virus (HIV) infection<br>Has known active tuberculosis (Bacillus tuberculosis)<br>Has a history or current evidence of any condition, therapy, or laboratory abnormality that might confound the results of the study, interfere with the participant's participation for the full duration of the study, or is not in the best interest of the participant to participate, in the opinion of the treating investigator<br>Has a known psychiatric or substance abuse disorder that would interfere with the participant's ability to cooperate with the requirements of the study<br>Is pregnant or breastfeeding or expecting to conceive or father children within the projected duration of the study, starting with the screening visit through 120 days after the last dose of study intervention<br>Has had an allogenic tissue/solid organ transplant	
Camrelizumab + apatinib	RESCUE (NCT03463876)	Patients with any active autoimmune disease or history of autoimmune disease, including but not limited to the following: hepatitis, pneumonitis, uveitis, colitis (inflammatory bowel disease), hypophysitis, vasculitis, nephritis, hyperthyroidism, and hypothyroidism, except for subjects with vitiligo or resolved childhood asthma/atopy. Asthma that requires intermittent use of bronchodilators or other medical intervention should also be excluded.<br>Concurrent medical condition requiring the use of immunosuppressive medications, or immunosuppressive doses of systemic or absorbable topical corticosteroids. Doses > 10 mg/day prednisone or equivalent are prohibited within 2 weeks before study drug administration.<br>More than one regimen.<br>Known history of hypersensitivity to any components of the SHR-1210 formulation, or other antibody formulation.	[49]

Known or occurrence of central nervous system (CNS) metastases or hepatic encephalopathy.

Patients with tumor burden ≥50% of the liver volume or received liver transplantation.

Patients with clinical symptoms of ascites.

Hypertension and unable to be controlled within normal level following treatment of anti-hypertension agents(within 3 months): systolic blood pressure > 140 mmHg, diastolic blood pressure > 90 mmHg.

Clinically significant cardiovascular and cerebrovascular diseases, including but not limited to severe acute myocardial infarction within 6 months before enrollment, unstable or severe angina, or coronary artery bypass surgery, Congestive heart failure (New York heart association (NYHA) class > 2), ventricular arrhythmia which need medical intervention.

Coagulation abnormalities (INR>2.0、PT>16s), with bleeding tendency or are receiving thrombolytic or anticoagulant therapy.

Previous digestive tract bleeding history within 3 months or evident gastrointestinal bleeding tendency, such as: esophageal varices, local active ulcerative lesions, gastric ulcer and duodenal ulcer, the ulcerous colitis, gastrointestinal diseases such as portal hypertension or resection of tumor with bleeding risk, etc.

Previous Arterial/venous thrombosis events within 3 months.

Proteinuria ≥ (++) and 24 hours total urine protein > 1.0 g.

Prior systemic chemotherapy, radiotherapy, immunotherapy, hormone therapy, surgery or target therapy within 4 weeks (Or 5 half-life of the drug, calculate the longer) before the study drug administration, or any unresolved AEs > Common Terminology Criteria for Adverse Events (CTCAE) Grade 1.

Active infection or an unexplained fever > 38.5°C during screening visits or on the first scheduled day of dosing.

History of immunodeficiency or human immunodeficiency virus (HIV) infection.

HBV DNA>2000 IU/ml（or 104copies/ml），HCV RNA>103copies/ml, HBsAg+ and anti-HCV+;

Patients with other malignant tumor (except cured skin basal cell carcinoma and cervical carcinoma).

Patients who has bone metastasis, has received Palliative radiotherapy (radiotherapy area > 5% marrow area).

Patients must not have had prior treatment with SHR-1210 or any other PD-L1 or PD-1 antagonists or apatinib.

Patients who may receive live vaccine during the study, or previous had vaccination within 4 weeks.

Any other medical, psychiatric, or social condition deemed by the investigator to be likely to interfere with a subject's rights, safety, welfare, or ability to sign informed consent, cooperate, and participate in the study or would interfere with the interpretation of the results.

Camrelizumab + apatinib	NCT03764293	Known hepatocholangiocarcinoma, sarcomatoid HCC, mixed cell carcinoma and lamellar cell carcinoma; other active malignant tumor except HCC within 5 years or simultaneously<br>Moderate-to-severe ascites with clinical symptoms<br>History of gastrointestinal hemorrhage within 6 months prior to the start of study treatment or clear tendency of gastrointestinal hemorrhage<br>Abdominal fistula, gastrointestinal perforation or intraperitoneal abscess within 6 months prior to the start of study treatment<br>Known genetic or acquired hemorrhage or thrombotic tendency<br>Thrombosis or thromboembolic event within 6 months prior to the start of study treatment<br>Cardiac clinical symptom or disease that is not well controlled<br>Hypertension that can not be well controlled through antihypertensive drugs<br>Factors to affect oral administration<br>History of hepatic encephalopathy<br>Previous or current presence of metastasis to central nervous system<br>HIV infection<br>Combined hepatitis B and hepatitis C co-infection<br>Be ready for or previously received organ or allogenic bone marrow transplantation<br>Interstitial lung disease that is symptomatic or may interfere with the detection and management of suspected drug-related pulmonary toxicity<br>Active known, or suspected autoimmune disease<br>Subjects with a condition requiring systemic treatment with either corticosteroids or other immunosuppressive medications within 14 days of first administration of study treatment<br>Use of potent CYP3A4 inducers or inhibitors within 2 weeks prior to the signature of ICF<br>Known history of serious allergy to any monoclonal antibody or targeted anti-angiogenic drug<br>Severe infection within 4 weeks prior to the start of study treatment<br>Palliative radiotherapy for non-target lesions to control symptoms is allowed, but it must be completed at least 2 weeks prior to the start of study treatment<br>Treatment of other investigational product(s) within 28 days prior to the start of study treatment	[53]
Avelumab + axitinib	VEGF Liver 100 (NCT03289533)	Prior systemic treatment for advanced HCC, including prior treatment with approved or investigational drugs.<br>Any prior locoregional therapy within 4 weeks and radiotherapy or surgical procedure within 2 weeks (4 weeks for major surgery) of enrollment.<br>Patients with known symptomatic brain metastases requiring steroids.<br>Presence of hepatic encephalopathy (ie, Child Pugh score of 2 or 3) and/or clinically relevant ascites (ie, Child Pugh score of 3).	[50]

		Presence of main portal vein invasion by HCC.<br>Any of the following within the 12 months prior to enrollment: myocardial infarction, severe/unstable angina, coronary/peripheral artery bypass graft, symptomatic congestive heart failure, LVEF less than LLN, clinically significant pericardial effusion, cerebrovascular accident, transient ischemic attack.<br>Active infection requiring systemic therapy except for hepatitis C virus (HCV) and hepatitis B virus (HBV).	
Cabozantinib + atezolizumab	COSMIC-312 (NCT03755791)	Known fibrolamellar carcinoma, sarcomatoid HCC or mixed hepatocellular cholangiocarcinoma.<br>Prior systemic anticancer therapy for advanced HCC including but not limited to chemotherapy, small molecule kinase inhibitors, and immune checkpoint inhibitors (ICIs). Subjects who have received local intratumoral or arterial chemotherapy are eligible; local anticancer therapy must be completed ≥ 28 days before randomization<br>Radiation therapy for bone metastasis within 2 weeks, any other radiation therapy within 8 weeks prior to randomization.<br>Known brain metastases or cranial epidural disease unless adequately treated with radiotherapy and/or surgery (including radiosurgery) and stable for at least 8 weeks prior to randomization.<br>Concomitant anticoagulation with oral anticoagulants	[51]
Camrelizumab + FOLFOX4/GEMOX	NCT03092895	Known fibrolamellar HCC; Prior malignancy active with the previous 5 years except for locally curable cancers that have been apparently cured.<br>Known or occurrence of central nervous system (CNS) metastases.<br>Ascites with clinical symptoms.<br>Known or evidence of GI hemorrhage within the past 6 months.<br>Known or occurrence of hemorrhage/ thrombus.<br>Known or evidence of abdomen fistula, gastrointestinal perforation, or abdominal abscess within the past 2 months.<br>Suffered from grade II or above myocardial ischemia or myocardial infarction, uncontrolled arrhythmias.<br>Grade III~IV cardiac insufficiency, according to NYHA criteria or echocardiography check: LVEF<50%.<br>Hypertension and unable to be controlled within normal level following treatment of anti-hypertension agents (systolic blood pressure > 140mmHg, diastolic blood pressure > 90 mmHg).<br>Factors to affect oral administration (such as patients unable to swallow oral medications, chronic diarrhea and ileus etc. situations evidently affect drug oral medication and absorption).<br>History of hepatic encephalopathy.<br>Known history of human immunodeficiency virus (HIV) infection.<br>Active infection or an unexplained fever > 38.5°C during screening visits.	[77]

		Has received a live vaccine within 30 days.<br>Prior or planning to organ transplantation including liver transplantation.<br>Interstitial lung disease that is symptomatic or may interfere with the detection and management of suspected drug-related pulmonary toxicity.<br>Proteinuria≥ 2+ or 24 hours total urine protein > 1.0 g.<br>Active known, or suspected autoimmune disease.<br>Subjects with a condition requiring systemic treatment with either corticosteroids (>10 mg daily prednisone equivalent) or other immunosuppressive medications within 14 days of first administration of study treatment. Inhaled or topical steroids, and adrenal replacement steroid doses > 10 mg daily. prednisone equivalent, are permitted in the absence of active autoimmune disease<br>Any loco-regional therapy to liver (included but not limited: resection, radiotherapy, TAE, TACE, TAI, RFA or PEI) within 4 weeks prior to study.<br>Prior therapy with anti-PD-1 or other anti-PD-1/anti-PD-L1 immunotherapy.<br>Known history of hypersensitivity to monoclonal antibodies or any components of the study drugs.<br>Treatment with anti-coagulation therapy(Warfarin or heparin) or anti-platelet therapy(aspirin at dose≥300mg/day, clopidogrel at dose≥75mg/day).<br>Pregnant or breast-feeding women.	
Tremelimumab + TACE or RFA	NCT01853618	Patients who have had standard of care chemotherapy, large field radiotherapy, or major surgery must wait 2 weeks prior to entering the study. For recent experimental therapies a 28 day period of time must elapse before treatment.<br>Patients who have undergone prior liver transplantation are ineligible.<br>Patients with known brain metastases will be excluded from this clinical trial<br>Uncontrolled intercurrent illness including, but not limited to, ongoing or active systemic infection, symptomatic congestive heart failure, unstable angina pectoris, cardiac arrhythmia (excluding insignificant sinus bradycardia and sinus tachycardia) or psychiatric illness/social situations that would limit compliance with study requirements.<br>History of chronic autoimmune disease (e.g., Addison's disease, multiple sclerosis, Graves disease, Hashimoto's thyroiditis, rheumatoid arthritis, hypophysitis, etc.) with symptomatic disease within the 3 years before randomization.<br>Dementia or significantly altered mental status<br>Diverticulitis (either active or history of) within the past 2 years.<br>Active or history of inflammatory bowel disease (colitis, Crohn's), irritable bowel disease, celiac disease, or other serious, chronic, gastrointestinal conditions associated with diarrhea. Active or history of systemic lupus erythematosus or Wegener's granulomatosis.	[82]

		Currently receiving immunosuppressive doses of steroids or other immunosuppressive medications (inhaled and topical steroids are permitted)<br>History of sarcoidosis syndrome<br>Patients should not be vaccinated with live attenuated vaccines within 1 month of starting Tremelimumab treatment.<br>Has a known history of Human Immunodeficiency Virus (HIV) (HIV 1/2 antibodies)<br>History of hypersensitivity reaction to human or mouse antibody products.<br>Pregnancy and breast feeding are exclusion factors<br>Patients with unhealed surgical wounds for more than 30 days.	
Pembrolizumab + TACE	NCT03397654	Has extrahepatic metastasis.<br>Prior TACE or systemic anticancer treatment for HCC.<br>Has any contraindication for TACE including porto-systemic shunt, hepatofugal blood flow, known severe atheromatosis.<br>Has history of bleeding within the 4 weeks preceding study enrolment.<br>Has hepatic encephalopathy.<br>Has ascites that is refractory to diuretic therapy.<br>Has documented occlusion of the hepatic artery or the main portal vein (segmental portal vein thrombosis does not represent exclusion criterion provided this does not contraindicate TACE).<br>Is currently participating and receiving therapy or has participated or is participating in a study of an IMP or used an investigational device within 4 weeks of the first dose of IMP.<br>Has a diagnosis of immunodeficiency or is receiving systemic steroid therapy or any other form of immunosuppressive therapy.<br>Has a known history of active Bacillus Tuberculosis (TB)<br>Hypersensitivity to Pembrolizumab or any of its excipients.<br>Has a known additional malignancy that is progressing or requires active treatment. Exceptions include basal cell carcinoma of the skin or squamous cell carcinoma of the skin that has undergone potentially curative therapy or in situ cervical cancer.<br>Has active autoimmune disease that has required systemic treatment in the past 2 years (i.e. with use of disease modifying agents, corticosteroids or immunosuppressive drugs).<br>Has known history of, or any evidence of active, non-infectious pneumonitis.<br>Has an active infection requiring systemic therapy.<br>Has a history or current evidence of any condition, therapy, or laboratory abnormality that might confound the results of the trial, interfere with the subject's participation for the full duration of the trial	[83]

	Has known psychiatric or substance abuse disorders that would interfere with cooperation with the requirements of the trial.<br>Is pregnant or breastfeeding, or expecting to conceive or father children within the projected duration of the trial, starting with the screening visit through to 120 days after the last dose of IMP.<br>Has received prior therapy with an anti-PD-1, anti-PD-L1, or anti-PDL2 agent.<br>Has a known history of Human Immunodeficiency Virus (HIV; HIV 1/2 antibodies).<br>Has received a live vaccine within 30 days of first dose of IMP administration.
